# Injectable decellularized cartilage matrix hydrogel encapsulating urine-derived stem cells for immunomodulatory and cartilage defect regeneration

**DOI:** 10.1038/s41536-022-00269-w

**Published:** 2022-12-22

**Authors:** Junfeng Zeng, Liping Huang, Huazhang Xiong, Qianjin Li, Chenyu Wu, Yizhou Huang, Huiqi Xie, Bin Shen

**Affiliations:** 1 Orthopedics Research Institute, Department of Orthopedics, West China Hospital, Sichuan University, Chengdu, Sichuan 610041 China; 2 Laboratory of Stem Cell and Tissue Engineering, Orthopedic Research Institute, Med-X Center for Materials, State Key Laboratory of Biotherapy, West China Hospital, Sichuan University, Chengdu, Sichuan 610041 China; 3 The First Department of Orthopedics, Affiliated Hospital of Zunyi Medical University, Zunyi, Guizhou 563006 China

**Keywords:** Trauma, Regeneration, Biomaterials - cells

## Abstract

Reconstruction of complex cartilage defects has remained a great challenge for tissue engineering due to the lack of stem cells and chronic inflammation within the joint. In this study, we have developed an injectable pig cartilage-derived decellularized extracellular matrix (dECM) hydrogels for the repair of cartilage defects, which has shown sound biocompatibility and immunomodulatory capacity both in vitro and in vivo. The dECM hydrogels can enhance the chondrogenic differentiation of human urine-derived stem cells (USCs). As shown by in vitro experiment, the USCs in the dECM hydrogels have survived, proliferated, and produced a mass of cartilage-specific extracellular matrix containing collagen II and aggrecan. And the USCs-laden dECM hydrogels have shown the capacity to promote the secretion of extracellular matrix, modulate the immune response and promote cartilage regeneration in the rat model for cartilage defect.

## Introduction

Articular cartilage defects caused by a sports injury, trauma, and cartilage degeneration are common in clinical practice^1–3^. Such defects are difficult to repair as it is difficult to regenerate avascular, aneural, and lymphatic tissues with complex structures to fulfill the unique mechanical demands^4,5^. On the other hand, it is important to enhance the repair and regeneration process in order to avoid or delay the cartilage defect to develop into osteoarthritis. Over the past decades, various strategies, including auto/allografts^6–8^, tissue-engineered materials, and stem cells, have been used to reconstruct the cartilage^9,10^. However, drawbacks such as secondary trauma caused by the autograft, the low survival rate of cells in the transplant material, and the formation of fibrocartilage within the defect have restricted their application^11,12^. New approaches to regenerate the damaged cartilage are therefore required.

In recent years, incorporating stem cells into biomaterials to promote cartilage regeneration has attracted much interest^13,14^. Adipose-derived stem cells (ADSCs)^15–17^, bone marrow mesenchymal stem cells (BMSCs)^18,19^ and synovial membrane mesenchymal stem cells (SM-MSCs)^20,21^ have been used to induce osteogenic, adipogenic, and chondrogenic differentiation in vivo. Despite the extensive effort and remarkable progress, the disadvantages of such cells, including invasive acquisition, limited proliferation capacity, and poor maintenance of their phenotype, are yet to be overcome^22^. As a novel type of MSCs, human urine-derived stem cells (USCs) possess the potential for robust proliferation and multi-potent differentiation with minimum ethical restriction^23,24^. Bharadwaj et al. have shown that the USCs can express chondrogenic lineage markers such as glycosaminoglycans (GAGs), Sox9, collagen II (COL-II), and aggrecan after 28 days of 3D culture in a chondrogenic medium^25^. Chen et al. showed that the USCs can differentiate into chondrocytes in vitro. Moreover, incorporating the USCs with hyaluronic acid could significantly promote neocartilage formation in a rabbit model for knee joint defect^7^. Nevertheless, report on the application of the USCs in cartilage tissue engineering is still scarce, and their chondrogenic capacity awaits further in vivo studies.

To provide a favorite microenvironment for stem cells, biomaterials should ideally possess sound biocompatibility, biodegradability, suitable mechanical strength, and plasticity. Biomaterials currently used for cartilage tissue engineering may be divided into two categories: (i) natural materials such as chitosan^1,26^, collagen^27^, gelatin^28^, and fibrin^29^, and (ii) synthetic materials such as polyethylene glycol (PEG)^30^, polycaprolactone (PCL)^31^, and polylactic acid (PLA)^32^. Synthetic materials usually have sound biomechanical strength, and their properties may be tailored by altering the composition of polymers. However, a major challenge for such materials is to achieve satisfactory tissue integration and differentiation as they are foreign to the body. Most natural biomaterials may overcome this as they are made of extracellular matrix components^33,34^. However, studies have reported that the use of such biomaterials may induce the host’s immune response and even the formation of granuloma and necrosis^3,35,36^. Recent studies have shown that decellularized matrix is free of the immunogenic cartilage cells and has preserved the bioactive components of extracellular matrix^37,38^. Among these, decellularized extracellular matrix (dECM) has a broad prospect for clinical applications. Dwikora et al. have reported that decellularized bovine cartilage scaffold sponge could promote the adhesion, proliferation, and chondrogenic differentiation of human bone MSC cells (hBM-MSCs) in vitro without the addition of any chondrogenic induction factors^39^. Li et al. also showed that decellularized pig cartilage matrix scaffolds combined with autogenous chondrocytes could induce the formation of neocartilage and better structural restoration 8 weeks after the transplantation in a rabbit model for knee articular cartilage defect^12^. Despite the great advances which have been made in promoting cartilage remolding, the dECM is mainly processed to films or powders, which have limited their applications in vitro and in vivo. Nevertheless, researchers have recently discovered that the dECM materials could be solubilized and processed into hydrogels without affecting the inherent bioactivity of the native matrix^38^. Furthermore, the dECM hydrogels can easily be injected in the form of pre-gel viscous liquid and polymerized at physiologic temperature into the form of hydrogel to match the shape of the defect. Based on this, we have chosen the dECM hydrogel as the cartilage implant biomaterial.

**Fig. 1:**
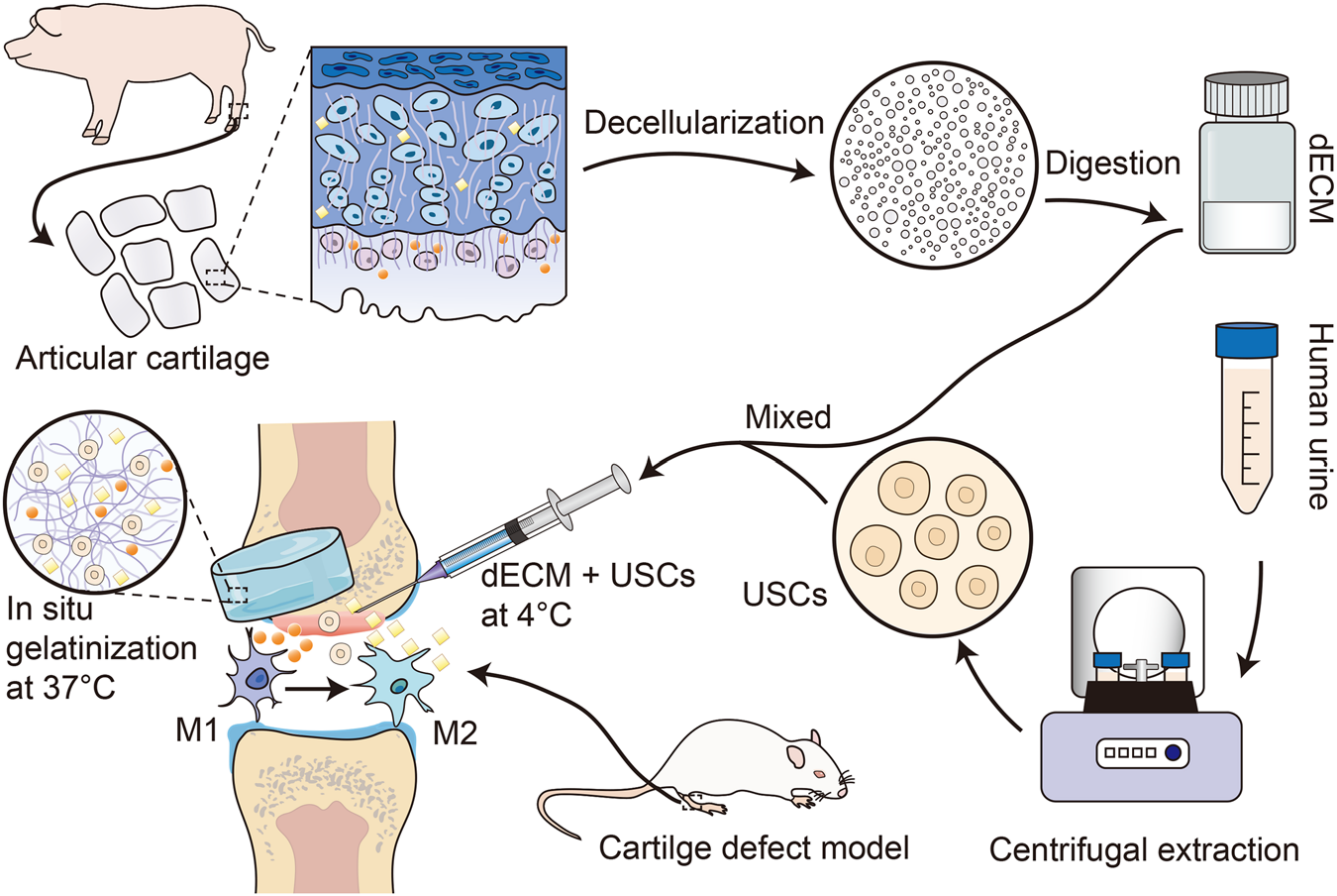
Graphical abstract of the study. Schematic illustrations of the injectable decellularized cartilage matrix (dECM) hydrogel encapsulating human urine-derived stem cells (USCs) for immunomodulatory and cartilage defect regeneration.

As an exogenous substance, a biomaterial implant may induce the host’s immune response and fibrosis at the defect site due to exacerbated inflammatory response^40–42^. Macrophages are known to play a critical role in cartilage repair and may be stimulated into M1 and M2 subgroups during cartilage damage^43^. Studies have shown that the M1 subgroup mostly secret proinflammatory cytokines, e.g., iNOS, IL-6, TNF-α, which may stimulate the body’s immune response to hamper the cartilage repair^44^. By contrast, M2 macrophages mainly secret anti-inflammatory IL-10, ARG-1, and CD206, which may attenuate the inflammatory response and promote cartilage repair^45^. Therefore, immunomodulatory capacity should also be considered during the development of the material. In a recent report, studies have also shown that some decellularized biomaterials could promote the transition of M1 macrophages into M2 macrophages^46^. Moreover, researchers have also shown that stem cells possessed strong immunomodulatory properties^47^. In this regard, we have developed an injectable hydrogel and combined it with the USCs to repair the knee articular cartilage defects (Fig. 1). The immunomodulatory capacity of the dECM hydrogels was also assessed.

## Results and discussion

### Characterization of the USCs

**Fig. 2:**
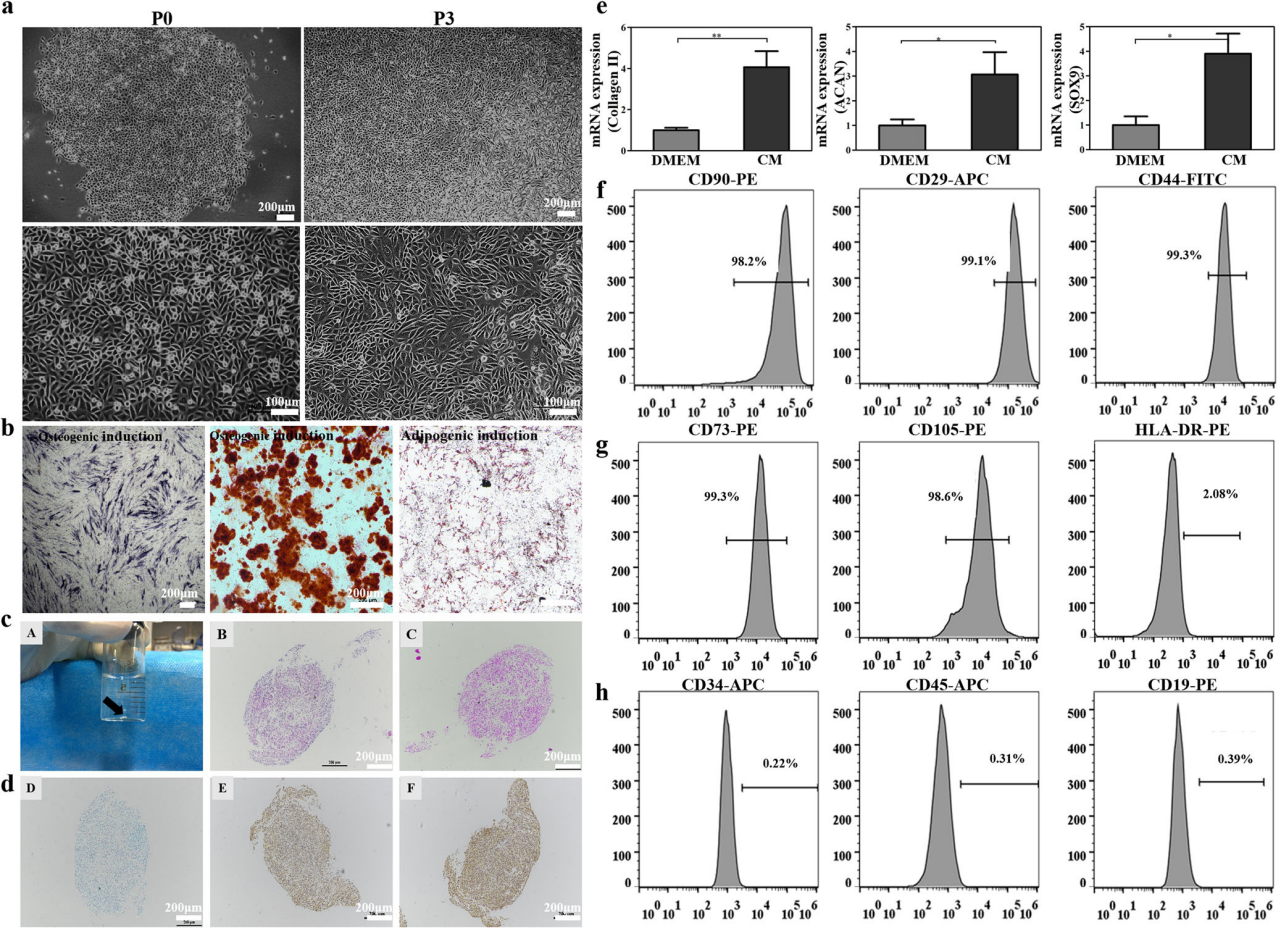
Characterization of human USCs. **a** Morphology and proliferation of the USCs. Scale bars = 100 and 200 μm. **b** Representative images of osteogenic-induced (by ALP and Alizarin Red staining) and adipogenic-induced (by Oil red O staining) with USCs. Scale bar = 200 μm. **c** The potential for chondrogenic differentiation of the USCs after 28 days of 3D chondrogenic differentiation in vitro: (A) Gross appearance; (B) H&E staining; (C) safranin O staining; (D) Alcian blue; (E) aggrecan; (F) collagen II. Scale bar = 200 μm. **e** The mRNA expression of chondrogenesis-related genes (Aggrecan, Sox9, and Collagen II) was quantified in the USCs after 28 days of culture. **f**–**h** Expression of surface markers of the USCs. DMEM (standard culture medium); CM (chondrogenic induction medium). ns, not significant; *P < 0.05, **P < 0.01, ***P < 0.001, * is the statistical difference compare with DMEM group. One-way ANOVA followed by the Tukey post hoc test was used. Each data point represented an average ± standard deviation, *n* = 4.

The USCs were successfully isolated from the human urine samples. As shown in Fig. 2a, the USCs have attached to the culture plate and displayed a rice-grain-like appearance after 2–3 days. They have further formed clones after 7 days without alteration in their morphology after many rounds of passages. After 10–12 days, the cell fusion rate has reached 80–90%, and the cells were passaged with a ratio of 1:2 or 1:3. As shown by the CCK-8 assay, the USCs have proliferated rapidly during the first 5 days but slowed down from 5 to 7 days and reached a plateau by the 7th day (Supplementary Fig. 1). The population doubling time of the USCs showed a trend similar to that by CCK-8 assaying (Supplementary Fig. 2), with the number of cells cultured for 3 days reaching approximately 40.93-fold compared with day 1. On days 5 and 7, the cells increased by approximately 65.68-fold and 71.82-fold, suggesting that the USCs possessed excellent proliferation capacity. Flow cytometry showed that they have expressed MSCs’ surface markers CD73, CD105, CD29, CD44, and CD90 (Fig. 2f–g) but not hematopoietic stem cells surface markers HLA-DR, CD19, CD34, and CD45) (Fig. 2g–h), which has fit the criteria for the identification of stem cells^48^. As shown in Fig. 2b, abundant blue precipitates by alkaline phosphatase (ALP) staining and red calcium nodules by Alizarin red staining (ARS) may be seen in the USCs cultured in the osteogenic induction medium. By Oil red O staining, red beads-on-a-string lipid droplets could be seen in the intracytoplasm. These have indicated that the USCs possess excellent proliferative, osteogenic, and adipogenic capacities. As shown in Fig. 2c-d, the USCs could form a cell pellet through 3D culture in a chondrogenic medium after 28 days. By H&E, Alcian blue, Safranine O staining, quantitative reverse transcription polymerase chain reaction (RT-qPCR), and immunohistochemistry analysis, a large quantity of extracellular matrix was stained by Safranine O, suggesting that masses of specificity cartilage GAG have been secreted by the USCs. By immunohistochemistry analysis, abundant COL-II and aggrecan may be seen in the cell sphere. RT-PCR analysis suggested that the COL-II, ACAN, and SOX9 expressions are upregulated after the USCs have formed the cell pellets by the 3D culture (Fig. 2e).

### Preparation and characterization of the dECM hydrogels

**Fig. 3:**
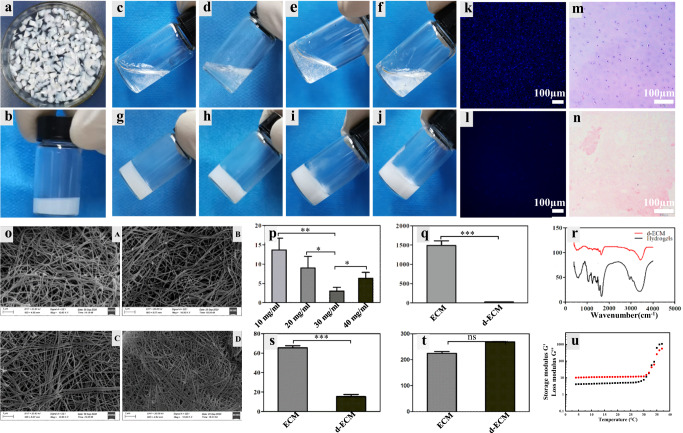
Characterization of the dECM hydrogel. **a** Gross appearance of the articular cartilage derived from the pig knee joint. **b** Representative images of the dECM hydrogels. **c**–**f** Various concentrations (10, 20, 30, 40 mg/mL, respectively) of the dECM hydrogels at 4 °C. **g**–**j** Various concentrations of the dECM hydrogels (10, 20, 30, 40 mg/mL, respectively) at 37 °C. **k**, **l** Representative images of articular cartilage characterization pre- and post-decellularization by DAPI staining. **m**, **n** Representative images of articular cartilage characterization pre- and post-decellularization by H&E staining. Scale bar = 100 μm. **h** Representative images of the dECM hydrogels (20×). Scale bar = 100 μm. **o** SEM images of various concentrations (10, 20, 30, and 40 mg/mL) of the dECM hydrogels. **q** DNA content in the ECM and dECM, which confirmed successful decellularization. **r** FTIR spectroscopy of the decellularized ECM (d-ECM) and dECM hydrogels. **s**, **t** GAG and Collagen II (COL-II) concentration of the ECM and dECM. **u** Rheological behavior of the dECM hydrogels. ns, not significant; *P < 0.05, **P < 0.01, ***P < 0.001, * is the statistical difference compare with ECM group. One-way ANOVA followed by the Tukey post hoc test was used. Each data point represents average ± standard deviation, *n* = 4.

An injectable dECM hydrogel was successfully developed by using pig articular cartilage as the raw material. As shown in Fig. 3c–j, the dECM hydrogels of various concentrations appeared as a liquid at 4 °C and hydrogel at 37 °C. The gelation time was determined by using a tube inversion method. The gelation time of the 10, 20, 30, and 40 mg/mL dECM hydrogels were 14, 9, 3, and 7 min, respectively (Fig. 3p). The articular cartilage was decellularized by lyophilization and enzyme digestion to remove the remaining cells which may induce an immune response to the implant. DAPI and H&E staining were carried out to observe the resident cells on the decellularized cartilage slice. As shown in Fig. 3k–n, masses of cell nuclei could be seen on the cartilage scaffold prior to the decellularization. The DNA, collagen, and GAGs contents of the decellularized cartilage scaffold were 23.5, 259.1 ± 12.45, 15.38 ± 2.22 ng/mg, respectively (Fig. 3q, s, t). Compared with untreated cartilage slices, the DNA content of the decellularized cartilage scaffold was significantly lower. These suggested that the collagen was successfully retained, and the GAGs were partially preserved by the lyophilization and enzyme digestion methods. By scanning electron microscopy (SEM) (Fig. 3o), all hydrogels exhibited porous structures, with the pore diameter decreasing along with the increased concentration of the dECM hydrogel. An equilibrium swelling method was then used to determine the water absorption capacity of the hydrogels (Supplementary Fig. 3). All the hydrogels have shown a higher swelling rate of around 1400%, and there was no obvious difference with various concentrations. Fourier Transform Infrared spectroscopy (FT-IR) was used to analyze the chemical structure of the dECM hydrogels (Fig. 3r). For dECM hydrogels, the peaks at 1590–1720 cm^−1^ and 1492–1590 cm^−1^ may be attributed to amido bonds, and the peak at 985–1140 cm^−1^ was assigned to the polysaccharide groups. As determined with a rheometer (Fig. 3u), the storage modulus of the dECM hydrogel was higher than the loss modulus, suggesting that the dECM solution was liquid at 4–33 °C. The storage modulus of the dECM hydrogels has reached 1065.39 ± 113.05 Pa at 37 °C, indicating that the dECM solution has been transformed into hydrogel and that the dECM hydrogels may be used as a tissue engineering material for cartilages.

### Biocompatibility of the dECM hydrogels

**Fig. 4:**
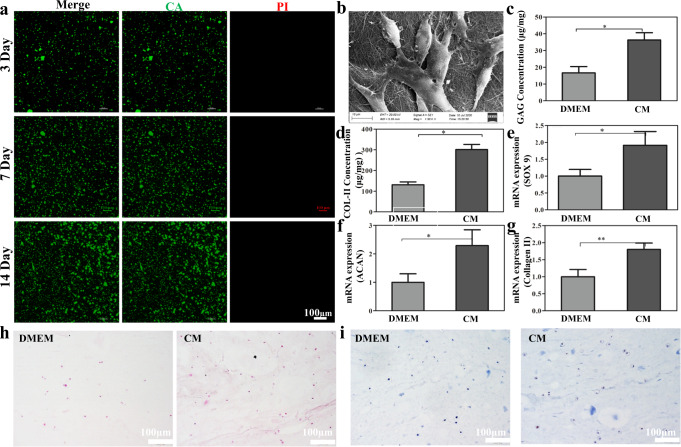
Biocompatibility assay of the dECM hydrogels. **a** Live&Dead cell staining of the USCs in the dECM hydrogel. **b** Morphology of the USCs cultured in dECM hydrogels. Scale bar = 100 μm. **c**, **d** The GAG and Collagen II (COL-II) concentrations of the USCs cultured in dECM with various mediums for 28 days. **e**–**g** Real-time PCR results of mRNA expression of SOX9, aggrecan (ACAN) and Collagen II in USCs cultured in dECM hydrogel with various mediums for 28 days. **h**, **i** H&E and Alcian blue staining was used to assessing the chondrogenic differentiation potential of the USCs cultured in the dECM hydrogel with various mediums for 28 days. DMEM (standard medium), CM (chondrogenic induction medium). Scale bar = 100 μm. ns, not significant; *P < 0.05, **P < 0.01, ***P < 0.001, * is the statistical difference compare with DMEM group. One-way ANOVA followed by the Tukey post hoc test was used. Each data point represents average ± standard deviation, *n* = 4.

Biocompatibility of the scaffold is critical for cartilage regeneration. To determine the biocompatibility of the dECM hydrogels, the USCs were mixed with dECM solution and incubated at 37 °C for gelation as well as for cell culture. As shown by CCK-8 and Live/Dead staining, the proliferation of the USCs was promoted in the dECM hydrogels. The USCs proliferated rapidly in the first 5 days and slowed down from 5 to 7 days, and reached a plateau on the 7th day (Supplementary Fig. 4). As shown by Live/Dead staining (Fig. 4a), most of the USCs in the dECM hydrogels exhibited good morphology after 14 days incubation with few dead cells in the hydrogels. By SEM, the USCs had a spindle-like or rice-grain-like morphology and were completely attached to the hydrogel (Fig. 4b). These indicated that the dECM hydrogels possess sound biocompatibility and may be used as the scaffold materials for facilitating the cartilage remolding.

### In vitro chondrogenic capacity of the dECM hydrogels

The in vitro chondrogenic induction properties of the scaffolds are critical for cartilage regeneration. To determine the chondrogenic induction properties of the dECM hydrogels, the USCs were mixed with dECM solution and incubated at 37 °C for gelation as well as cultured in the standard culture medium or chondrogenic induction medium for 28 days. As shown by Fig. 4h, i, H&E staining has revealed typical chondrocytes and lacunae architecture in the dECM hydrogels cultured in the standard or chondrogenic induction medium, whilst Toluidine blue staining showed considerable blue extracellular matrix structure in the hydrogel’s indicative of cartilage-specific extracellular matrix. Taken together, the USCs seeded in the dECM hydrogels possessed sound chondrogenic capacity with the standard or chondrogenic induction medium. To confirm the chondrogenic differentiation of the USCs in the dECM hydrogels, Hydroxyproline assay Kit, Blyscan™ Glycosaminoglycan Assay, and RT-qPCR assays were employed to determine the protein and mRNA expression of chondrogenesis-associated genes. The content of GAGs and COL-II has remarkably increased in the group treated within the chondrogenic induction medium compared with those cultured in standard medium (6.3 ± 7.5 vs. 16.7 ± 6.5, *P* < 0.001; 301.0 ± 44.3 vs. 131.0 ± 24.6, *P* < 0.05) (Fig. 4c, d). The expression levels of chondrogenic marker genes ACAN, SOX9, and COL-II have significantly increased in the group treated with the chondrogenic induction medium compared with the standard culture medium (Fig. 4e-g). The above results suggested that treating the USCs in the dECM hydrogels with a chondrogenic induction medium can confer them with great chondrogenic capacity.

### In vitro and in vivo immunomodulatory effect of the dECM hydrogels

**Fig. 5:**
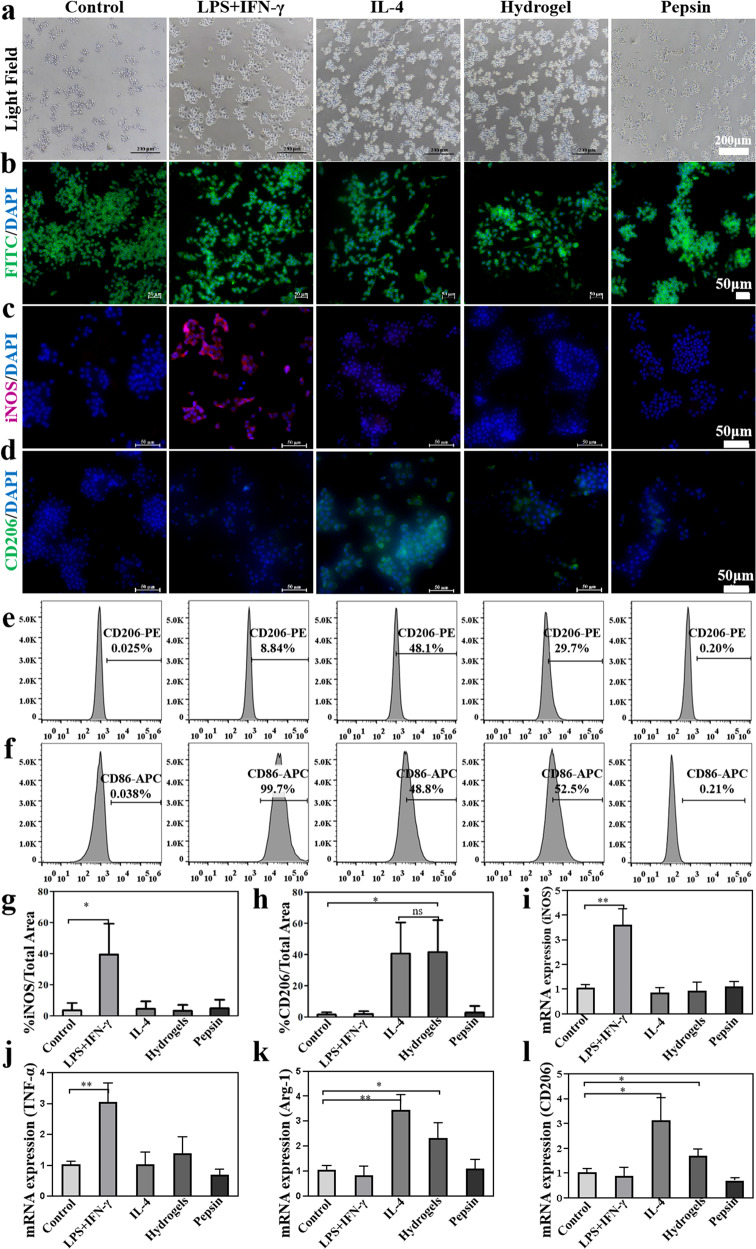
Raw 264.7 was co-cultured with dECM hydrogel in transwell and the immunomodulatory capacity assessment. **a** Bright-field image of the RAW264.7 cells treated with various stimuli for 48 h. Scale bar = 200 μm. **b** Morphology of the cells stained with FITC Phalloidin for the cytoskeleton (green) and DAPI for the nucleus (blue) after 48 h. Scale bar = 50 μm. **c**, **d** Immunofluorescence of iNOS and CD206 expression treated by different groups. Scale bar = 50 μm. **e**, **f** Representative images of surface markers CD86 and CD206 of RAW264.7 analyzed by flow cytometry. **g**, **h** Semi-quantitative analysis of immunofluorescence staining results. **i**–**l** The expression of inflammatory cytokines iNOS, TNF-α, ARG-1, and CD206 was detected by RT-qPCR. ns, not significant; *P < 0.05, **P < 0.01, ***P < 0.001, * is the statistical difference compare with Control group. One-way ANOVA followed by the Tukey post hoc test was used. Each data point represents average ± standard deviation, *n* = 4.

As an exogenous substance, a biomaterial implant may activate the immune response and cause fibrosis at the defect site due to dysregulated inflammatory response. Therefore, to improve the repairing and regenerative effect of the biomaterials, the immunomodulatory capacity should be considered^49^. Of their high plasticity, macrophages play a critical role in the host’s immune response to the biomaterials. In the present study, RAW264.7 macrophage cells have been used to assess the immunomodulatory effect of the dECM hydrogels^50^. Macrophages can be polarized into M0 (resting macrophages), M1 (classically activated), and M2 (alternatively activated) phenotypes under different conditions^51^. It has been reported that anti-inflammatory M2-like macrophages can increase the anabolism and reduce the catabolism of the cartilage, thereby promoting its regeneration^52^. To assess the influence of the dECM hydrogels on the macrophages, we have imitated the morphology of the macrophages with various stimuli (to culture M0 macrophages with a standard medium, M1 macrophages with 100 ng/ml LPS + 20 ng/ml IFN-γ, and M2 macrophages with 20 ng/ml IL-4, Macrophages in the experimental group were treated by dECM hydrogels, positive control group were treated by pepsin (The effect of pepsin addition during dECM digestion was excluded). As shown in Fig. 5a, b, the majority of M0 macrophages had a round shape, the M1 macrophages appeared more spherical with pseudopodia, whilst the M2 macrophages had a slenderer spindle shape. The macrophages treated by the dECM hydrogels were more like the M2 macrophages, whilst those treated by pepsin resembled the M0 macrophages. The above results have tentatively indicated that the dECM hydrogels have the potential to induce M0 polarization of M2 macrophages. Macrophage-specific markers iNOS (M1) and CD206 (M2) were detected to identify phenotypes of macrophages by immunofluorescence staining (IFS) (Fig. 5c, d). The M1 phenotypes groups and M2 phenotypes groups have specifically expressed iNOS and CD206, respectively. Compared with the control group, the expression of iNOS was remarkably reduced, and CD206 has markedly increased in the dECM hydrogels groups. However, the expression of iNOS and CD206 showed no obvious changes in the pepsin groups. The results of the semi-quantitative analysis also conformed to the above results (Fig. 5g, h). By flow cytometry (Fig. 5e, f), the M2 and the dECM hydrogels groups were highly positive (48%, 29.7%) for CD206 and moderately positive (48.8%, 52.5%) for CD86 expression. The M1 groups showed high expression of CD86 and low expression of CD206. As shown by RT-qPCR, the expression of pro-inflammatory cytokines iNOS, and TNF-α was markedly downregulated by the dECM hydrogels (Fig. 5i, j). Meanwhile, the pro-inflammatory of CD206 and ARG-1 were obviously up-regulated (Fig. 5k-l). The above results suggested that the dECM hydrogels can induce polarization of M0 into M2 macrophages, which can favor cartilage regeneration. The immunomodulatory capacity of the dECM hydrogels was further investigated in vivo. As shown in Supplementary Fig. 5A, and the appearance of dECM hydrogels showed no obvious difference at both times points 7 and 14 days after the implantation, the surface of subcutaneous tissues around the dECM hydrogels was smooth without granulation tissue hyperplasia, and new vessels formation was noted. H&E staining was further employed to evaluate the immunomodulatory capacity of the dECM hydrogels. As shown in Supplementary Fig. 5B, obvious inflammatory cell infiltration around the dECM hydrogels was noted on the 7th day. After 14 days, the inflammatory cells were reduced, and the structure of hydrogels has become sparse, suggesting that the dECM hydrogels have gradually degraded and absorbed. IFC analysis was applied to estimate the immune response after the dECM hydrogels implantation (Supplementary Fig. 5C and Supplementary Fig. 5D). CD86 staining results revealed numerous yellow-brown macrophages on the 7th day, which were gradually decreased during the following 14 days. By CD206 staining, few positively stained cells were noted on the 7th day, though there is a large number of positively stained cells after 14 days. These indicated that the dECM hydrogels mainly induced macrophage polarization to M1 macrophages in the early stage and promoted the transformation of the M1 macrophages into the M2 macrophages in the middle stages. These indicated the dECM hydrogels may facilitate tissue repair.

### In vivo cartilage regeneration capacity of the USCs-laden dECM hydrogels

**Fig. 6:**
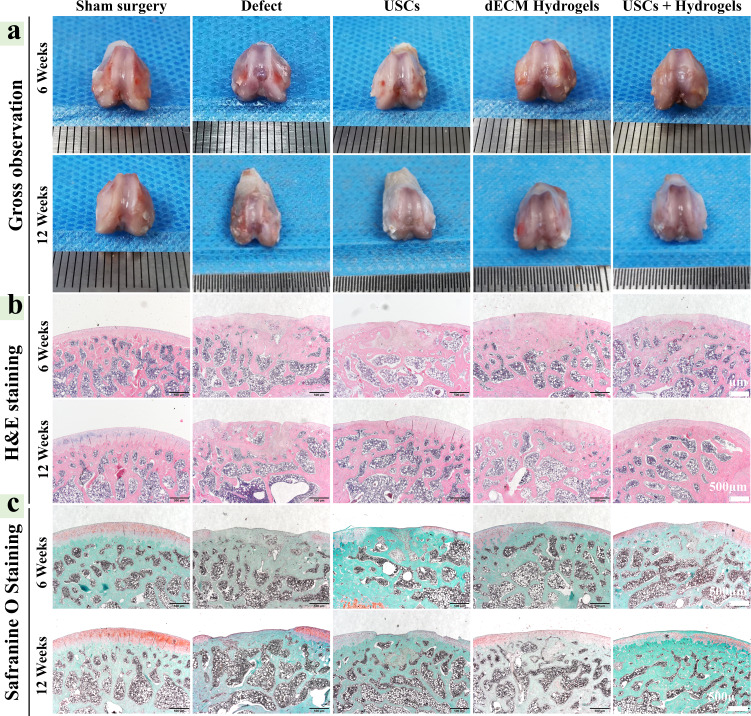
In vivo cartilage regeneration of cartilage defects treated with the USCs, dECM hydrogels, or USCs-laden dECM hydrogels. **a** Gross appearance of the cartilage at 6 and 12 weeks after the injection. **b** Representative H&E staining images of rat knee joints. Scale bar = 500 μm. **c** Safranin O/Fast Green staining of rat knees 6 and 12 weeks after the hydrogel injection (Scale bar = 500 μm).

**Fig. 7:**
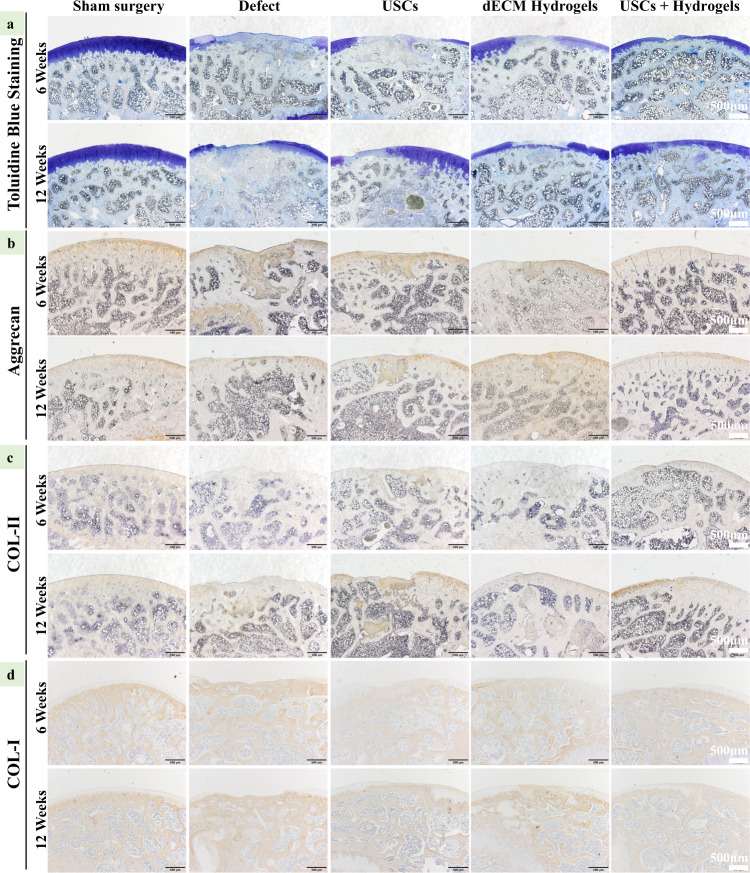
Expression of extracellular matrix proteins in the cartilage defect 6 and 12 weeks after the injection of the hydrogels. **a** Toluidine blue staining of the cartilage. **b** Immunochemistry for aggrecan expression in the defect area (Scale bar = 500 μm). **c** Immunochemistry for collagen II expression in the defect area. Scale bar = 500 μm. **d** Immunochemistry for collagen I expression in the defect area. Scale bar = 500 μm.

A rat model for full-thickness cartilage defect was constructed to assess the compatibility and potential of chondral regeneration of the hydrogels in vivo (Supplementary Fig. 6). As shown in Fig. 6a, compared with other groups, the USCs-laden dECM group showed well-integrated and newly regenerated hyaline cartilage-like tissues 12 weeks after the implantation, whilst the untreated group only showed rough surface, few neo-tissue and poor chondral regeneration. The dECM hydrogels alone could enhance the repair by filling the cartilage defect to promote chondral regeneration. By H&E, Safranine O, and Toluidine Blue staining, the untreated defects displayed a rough surface, formation of granulation tissue, as well as poor chondral regeneration at 6 and 12 weeks. The USCs groups and dECM hydrogels groups also formed some new soft and bony tissues at 6 and 12 weeks, whilst the USCs-laden dECM hydrogel groups showed smoother surfaces, numerous chondroid tissues, and chondrocytes formation (Fig. 6b). As shown by Figs. 6c and 7a, no obvious Safranin-O and Toluidine Blue staining were noted in the defect area of the untreated groups, while strong Safranin-O and Toluidine Blue staining in the defect area of USCs-laden dECM hydrogels group suggested abundant GAG expression. On the other hand, the USCs and dECM hydrogels groups were partly positive, suggesting that the content of GAGs in the USCs-laden dECM hydrogels group was much higher compared with the USCs groups and dECM hydrogels groups. The USCs-laden dECM hydrogels group displayed a well-integrated and orderly continuous structure between cartilage and subchondral bone, while the USCs and dECM hydrogels groups had a deranged structure between the cartilage and subchondral bone. By immunohistochemistry analysis, compared with other groups, the regenerated cartilage tissues in the USCs-laden dECM hydrogels group showed higher expression of type II collagen and aggrecan but lower expression of type I collagen at 6 and 12 weeks (Fig. 7b–d). Results of the quantitative analysis showed a trend similar to that by immunohistochemistry staining that the USCs-laden dECM hydrogels promoted the expression of type II collagen and aggrecan while inhibiting the expression of type I collagen (Supplementary Fig. 7). According to the International Cartilage Repair Society (ICRS) macroscopic scoring system, the regenerated cartilage in the Sham surgery, defect, USCs, dECM hydrogels, and USCs-laden dECM hydrogels groups had scored 0.2 ± 0.4, 8.8 ± 2.3, 5.5 ± 2.2, 5.3 ± 2.6, and 4.5 ± 2.0 at 6 weeks, and 0.3 ± 0.5, 7.5 ± 1.9, 4.7 ± 1.6, 3.3 ± 1.2, and 2.5 ± 1.0 at 12 weeks, respectively (Supplementary Fig. 8). The total score of defects treated with the USCs-laden dECM hydrogels was significantly higher than the untreated groups, suggesting that the USCs-laden dECM hydrogels can facilitate cartilage regeneration in vivo and may be used as scaffold materials for cartilage remolding.

## Methods

### Isolation and culture of human urine-derived stem cells (hUSCs)

The hUSCs were obtained from healthy male adult donors aged 23 and 27 with a previously described method^23,53^. This study has been approved by the Ethics Committee for Biomedical Research, West China Hospital of Sichuan University (2021–1066), with written informed consent obtained from all participants. To 200 mL urine sample, 1% of penicillin and streptomycin were added and centrifuged at 400 × *g* for 10 min. The cell pellet was resuspended in 25 mL of phosphate-buffered saline (PBS) and centrifuged again for 10 minutes at 400 × *g*. The procedure was repeated once, and then, cells were seeded in 25 T flasks with culture medium comprised of keratinocyte serum-free medium, DMEM-HG, 5% fetal calf serum (FBS), 1% penicillin and streptomycin, and other supplements, including epidermal growth factor, bovine pituitary extract, hydrocortisone, transferrin, bovine insulin, adenine, and 3,3,5-triiodo-L-thyromine^54^. The cells were incubated in 5% CO_2_ at 95% humidity and 37 °C. The medium was replaced every three days. Once they reached subconfluency, the cells were passaged by using trypsin. The hUSCs from passage 3 were used for the subsequent experiment, with their morphology captured with a microscope imaging system. To evaluate their proliferation, the hUSCs were seeded into a 96-well plate and incubated in 100 μL of culture medium at 37 °C and 5% CO_2_. The viability of the cells was assessed on days 1, 3, 5, 7, and 9 with a CCK-8 assay (Life Technologies, USA). At each time point, 10 μL of CCK-8 reagent was added to each well, and the optical density was measured with a spectrophotometer at a wavelength of 490 nm. A CyQUANT® Cell Proliferation Assay Kit was used to evaluate the population doubling time of the USCs.

### Flow cytometry analysis

At passage 3, the hUSCs were harvested by using trypsin-EDTA. 1 × 10^6^ hUSCs were resuspended in PBS and incubated for 30 min at 4 °C in the darkness with monoclonal antibodies CD34-APC (Mouse, 1:1000, 555821), CD73-PE (Mouse, 1:500, 550257), CD105-PE (Mouse, 1:1000, 561443), HLA-DR-PE (Mouse, 1:1000, 335813), CD45-APC (Mouse, 1:1000, 347463), CD29-APC (Mouse, 1:500, 559883), CD19-PE (Mouse, 1:500, 340364), CD90-PE (Mouse, 1:500, 561558), CD44-FITC (Mouse, 1:500, 555478). Subsequently, the cells were washed and resuspended with 400 μL of PBS and analyzed with a FACScan Flow Cytometry Analyzer (Becton Dickinson, USA).

### Multilineage differentiation potential of the hUSCs

The potential of the hUSCs for osteogenic and adipogenic differentiation was verified by two-dimensional (2D) plate induction culture, and their chondrogenic differentiation ability was verified by a three-dimensional (3D) pellet formation experiment as previously described^24,55^. For the 2D induction culture, the hUSCs were seeded at a density of 5 × 10^4^ cells/well in 6-well plates in a culture medium. When the cells reached 80% confluence, the medium was replaced with a specific differentiation medium containing 10% FBS, 1% penicillin and streptomycin, 10 mM β-glycerophosphate, 50 μg/mL ascorbic acid and 0.2 μM dexamethasone. The medium was replaced every 3 days. Alkaline phosphatase (ALP; Sigma) staining was carried out after 14 days of osteogenic induction. Alizarin red S (ARS; Sigma) staining was used to assess the mineralization of the hUSCs after 21 days of osteogenic induction. Oil red O (ORO; Sigma) staining was performed to evaluate the adipogenic ability after 14 days of adipogenic induction.

After 28 days of chondrogenic induction, H&E, Alcian blue (Sigma), Oil red O staining, immunohistochemistry staining (Aggrecan and COL-II), and RT-qPCR were carried out. Chondrogenesis-related primary antibodies have included Aggrecan (Agg; mouse, Abcam, 1:200, ab3773) and COL-II (rabbit, Abcam, 1:200, ab34712). RT-qPCR was carried out to determine the expression of chondrogenesis-related genes, including COL-II, SOX9, and ACAN^26^, with GAPDH as the internal control. All primers were synthesized by Qinke Biotech (Shanghai, China) (Supplementary Table 1).

### Preparation and characterization of the dECM hydrogels

#### Decellularization

Fresh knee joints from adult pigs were purchased from a local abattoir. Articular cartilage from the knee and hip joints was carefully removed with scalpels^12^. The cartilage was washed twice in PBS and stored at −80 °C. To make cartilage slices, the samples were placed in a freeze-dryer refrigerator lyophilization for 24 h to remove the residual water. A ball mill instrument (Anton Paar) was further applied to pulverize the cartilage. The cartilage powder was packed into 50 mL centrifuge tubing and added with Tris-HCL buffer (pH 8.0) as well as triton X-100 (1% v/v), and placed on a shaker table for 24 h at 4 °C. The centrifuge tubing was then subjected to 400 × *g* centrifugation with two reciprocating washes with PBS, followed by adding 50 U/mL DNase-I solutions (Sigma-Aldrich) and 20 µg/mL RNase-I solution (Sigma-Aldrich) for the digestion. The digested powder was washed with PBS three times, added with Trypsin-EDTA solution, and placed on a shaker table for 12 h at 4 °C. The digested cartilage power was then removed from the 50 mL centrifuge tube and frozen and lyophilized. The cartilage was cryoground into a fine powder with a freezer mill and lyophilized overnight.

#### Synthesis of the dECM hydrogels

The ECM powder was mixed in 0.1 M HCl at a concentration of 50 mg/mL. Pepsin was added at a concentration of 1 mg/mL, and the solution was stirred at 200 × *g* for 2 days at 4 °C. The solution was neutralized to physiological pH by adding 1 M NaOH at 4 °C. The solubilized ECM powder (SECM) was incubated at 37 °C for gelation. The hydrogel gelation time was determined with a tube inversion method. The dECM pre-hydrogel mixture of various concentrations was prepared with the components listed in Supplementary Table 2.

#### Histological and biochemical evaluations of the dECM hydrogels

H&E and DAPI staining were employed to observe the residue of cells on the dECM hydrogels and decellularized cartilage slices. The GAG, DNA, and collagen contents of decellularized cartilage slices and dECM hydrogels were quantified with Blyscan™ Glycosaminoglycan Assay (Biocolor), Picogreen dsDNA Quantitation Reagent (Invitrogen, USA), and the hydroxyproline assay kit (Jiancheng, Nanjing, China), respectively, by following the manufacturers’ instructions.

#### Characterization of the dECM hydrogels

The scaffolds were observed with scanning electron microscopy (SEM; EVO MA 10/LS 10, Carl Zeiss AG, Germany). The swollen hydrogels were quickly frozen at −80 °C, freeze-dried in a vacuum at −50 °C for 2 days, and cut and placed on aluminum stubs sputtered with gold for 60 s. The swelling kinetics of the hydrogels of various concentrations was tested at room temperature over a period of 2 days. The dried hydrogels were weighed before the test and immersed in 10 mL of PBS. At each time point, the excess surface water of the samples was removed before weighing. For the hydrogels, the swelling ratio (Qt) was calculated with the equation: Qt = [(Wt-W0)/W0] × 100% (Wt: the weight of the hydrogel at time t), while the equilibrium swelling ratio (Qeq) was calculated as Qeq = [(We- W0)/W0] × 100% (W0: the weight of the dry hydrogel at t = 0; We: the weight of a swollen hydrogel at equilibrium). FT-IR was used to delineate the chemical structure of the hydrogels. The rheological properties of the dECM hydrogels were determined with a rheometer (DHR-1, USA). A plate-plate geometry with a diameter of 40 mm and plate-to-plate distance of 1 mm was used in all tests. The SECM was dropped on the plate, and the mechanical spectra were recorded at a constant frequency of 0.159 Hz; the temperature dependence of storage modulus (*G*′) and loss modulus (*G*′′) were measured with temperature scan ranging from 4 °C to 37 °C. The rheological behavior of the dECM hydrogel was tested four times.

### Biocompatibility of the dECM hydrogels

The USCs from passage 3 were harvested by using 0.25% trypsin, resuspended in DMEM solution to a final concentration of 1 × 10^5^ cells/ml, and mixed with the SECM. The USCs/SECM mixed solution was added to 24-well plates to form hydrogels and incubated in 5% CO_2_ at 95% humidity and 37 °C. The culture medium was replaced every 2 days. After 3 days of culture, the USCs-laden hydrogels were stained using the live/dead staining kit [Calcein-AM (Sigma-Aldrich, 1 μg/mL) and propidium iodide (PI, Sigma-Aldrich, 1 μg/mL)] by following the manufacturer’s instructions. The stained USCs-laden hydrogels were observed under a confocal laser scanning microscope (Nikon, Japan). The proliferation of the USCs in the dECM hydrogels was determined with a CCK-8 assay. SEM was further carried out to estimate the attachment of the USCs to the dECM hydrogels. Prior to the SEM, the samples were co-cultured for 3 days, fixed with 4% paraformaldehyde, and dehydrated in ethanol with increasing concentrations. Subsequently, the samples were dried with a critical point dryer and sputtered with gold.

### The chondrogenic ability of the USCs-laden hydrogels in vitro

The USCs from passage 3 were harvested by using 0.25% trypsin, resuspended in DMEM solution to a final concentration of 1 × 10^7^ cells/ml, and mixed with the SECM. The USCs/SECM mixture was added to 24-well plates to form the hydrogels, which were then incubated in 5% CO_2_ at 95% humidity and 37 °C. After incubated for one day, the culture medium was replaced with osteogenic medium containing 50 μg/mL ascorbic acid, 10 ng/ml TGF-β3, 0.1 μM dexamethasone, 40 μg/mL-proline, 100 μg/mL sodium pyruvate and 1% ITS every 2 days. After 28 days of chondrogenic induction, the USCs-laden dECM hydrogel was fixed with 10% paraformaldehyde and analyzed by H&E and Alcian blue staining. Expression levels of COL-II and GAGs proteins and the mRNA of COL-II, ACAN, and SOX9 were analyzed.

### In vitro immunomodulatory capacity of the dECM hydrogels

In this study, RAW264.7 cells were used to verify the immunomodulatory capacity of the dECM hydrogels. The cells (1 × 10^4^ cells/well) were incubated in the Transwell with 24-well plates and divided into the Control group (M0 macrophages cultured with standard medium); M1 positive control group (M0 macrophages treated by 100 ng/ml LPS + 20 ng/ml IFN-γ); M2 positive control group (M0 macrophages treated by 20 ng/ml IL-4); dECM hydrogel group [M0 macrophages were incubated on the lower layer of Transwell with 24-well plates, and dECM hydrogel was incubated on the upper layer of Transwell with 24-well plates (30 mg/mL)]; Pepsin group [M0 macrophages were incubated on the lower layer of Transwell with 24-well plates, and pepsin (1 mg/mL) was incubated on the upper layer of Transwell with 24-well plates]. After culturing for 48 h, the cells were observed under an optical microscope, and the RAW264.7 cells were fixed by 4% paraformaldehyde for the stain of the cytoskeleton and nucleus. The fixed cells were washed with PBS twice to remove the residue paraformaldehyde, permeabilized by using 1% Triton X-100 for 5–8 min and washed twice with PBS. Subsequently, the cells were incubated with 5 μg/mL FITC Phalloidin for 40 min in the darkness to stain the cytoskeletons and washed twice with PBS. Thereafter, 5 μg/mL DAPI was added to stain the nuclei for 8–10 min at room temperature. Images were recorded with a fluorescence microscope. The phenotypes of the macrophages were further identified by IFS. Briefly, the fixed RAW264.7 cells were washed with PBS twice, treated with 1% Triton X-100 for 30 min at 37 °C, blocked with 1% bovine serum albumin at 37 °C for 1 h, and incubated with primary anti-iNOS (Rabbit, 1:200, ab178945) and anti-CD206 antibodies (Rabbit, 1:200, ab125028) at 4 °C overnight. After rinsing in PBS for three times, the secondary anti-mouse IgG antibody was added to the samples and further incubated for 1 h at 37 °C. Flow cytometry was also used to analyze the expression ratio of the macrophage-specific markers CD86 (M1) and CD206 (M2). RAW264.7 cells (1 × 10^5^/well) were treated by different groups for 48 h. The cells were then digested with 0.25% trypsin and harvested. The cells were washed with PBS twice, treated with CD86 and CD206 serum-free DMEM for 20–30 min at 37 °C, and washed with PBS and centrifugated. Thereafter, the cells were resuspended in 400 μL PBS and placed on ice. Intracellular fluorescent signals were detected by flow cytometry (Becton Dickinson, USA). The inflammatory gene expression levels (iNOS, CD206, TNF-α, ARG-1) were further estimated by RT-qPCR. GAPDH was used as a control. All primers were synthesized by Qinke Biotech (Shanghai, China) (Supplementary Table 3).

### The immunomodulatory capacity of the dECM hydrogels in vivo

Sprague-Dawley (SD) rats weighing 200–250 g were purchased from Chengdu Dashuo Experimental Animal, Co., Ltd. The rats were anesthetized with isoflurane. After aseptic preparation, 0.4 mL dECM hydrogel was subcutaneously injected into the back of the rats. All rats were monitored during recovery from anesthesia and sacrificed after 7 and 14 days. The samples were collected for gross observation and immunomodulatory analysis. H&E and immunohistochemistry staining (CD86, CD206) were employed to determine the immune reaction induced by the dECM hydrogels.

### Cartilage-defect animal model

The USCs (10^6^ cells/50 μL) were thoroughly mixed with the dECM (30 mg/mL), and placed in 4 °C refrigerators. The USCs-laden dECM hydrogel was gently transferred into a 1 mL syringe and placed on the ice.

Animal caring and experimentation protocols were approved by the Animal Care and Use Committee of Sichuan University (Ethics Approval Number: 2020226A). Healthy male SD rats (12 weeks) weighing 200–250 g were anesthetized through isoflurane, and the knee joint was opened through a medial parapatellar approach. The patella was dislocated laterally to expose the femoropatellar groove. With a stainless-steel punch, a full-thickness cylindrical cartilage defect (2 mm in diameter and 0.5 mm in depth) was created on the trochlear groove of the hind limbs (Supplementary Fig. 6A). Normal saline was used to wash the joint cavity and wound site. The rats were divided into five groups: sham surgery, defects without treatment (non-treated group); defects filled with the USCs (10^6^ cells/50 μl); defects filled with the dECM hydrogels only (50 μL); defects filled with USCs-laden dECMS hydrogels (10^6^ cells mixed with dECM/50 μL, Supplementary Fig. 6B and Supplementary Fig. 6C). After the surgery, the animals were returned to cages without joint immobilization and were sacrificed in 6 and 12 weeks, with the knee joint. samples collected for gross observation and cartilage regeneration analysis.

### Histological evaluation

The tissue samples were fixed with 4.0% paraformaldehyde for a week and decalcified in 10% EDTA solution for 2 weeks. After decalcification, the cartilage tissues were embedded into the paraffin, sectioned at a 5-μm thickness in the sagittal direction of the artificial defect, and subjected to H&E staining for morphological evaluation, Saf-O/Fast Green and alcian blue staining for Glycosaminoglycan (GAG) distribution, and Aggrecan (Mouse, 1:200, ab3778), COL-I (Rabbit, 1:200, ab254113) and Col-II (Rabbit, 1:200, ab34712) for cartilage-specific phenotype evaluation. The slides were observed under optical microscopy (BX63, Olympus, Japan). ImageJ software was used to quantify the results of immunohistochemical staining (Aggrecan, COL-I, and Col-II). The ICRS visual histological score of defects was blindly evaluated by three independent researchers.

### Statistical analysis

All data were presented as mean ± standard deviation and analyzed by one-way ANOVA with a post hoc test. *P* < 0.05 was considered statistically significant with * representing 0.01 < *P* < 0.05, ** representing 0.001 < *P* < 0.01, and *** representing *P* < 0.001.

## Conclusion

In summary, we have developed an injectable dECM hydrogel with sound biocompatibility by using the USCs and RAW264.7 cells. The USCs-laden dECM hydrogels have shown significant chondrogenic and immunomodulatory capacity, allowing the hydrogel to modulate the inflammatory environment and promote cartilage regeneration both in vitro and in vivo. The USCs-laden dECM hydrogels may therefore provide a promising biomaterial for cartilage regeneration.

## Supplementary information

Supplementary material

## Data Availability

The datasets generated during and/or analyzed during the current study are available from the corresponding author upon reasonable request.
