# Supplementary material for: Injectable decellularized cartilage matrix hydrogel encapsulating urine-derived stem cells for immunomodulatory and cartilage defect regeneration
Source: NPJ Regen Med. 2022 Dec 22;7:75. doi: 10.1038/s41536-022-00269-w (PMC9780205; doi:10.1038/s41536-022-00269-w)
Supplement: Supplementary file 1 — Supplementary material [file 41536_2022_269_MOESM1_ESM.pdf]

Supplementary Table 1. Primer pairs used in the qRT-PCR.

Gene	Forward primer (5’-3’)	Reverse primer (5’-3’)
COL2A1	CAACCAGGACCAAAGGGACA	ACCTTTGTCACCACGATCCC
SOX9	GAAGGACCACCCGGATTACA	GCCTTGAAGATGGCGTTGG
ACAN	GAAGGGCGAGTGGAATGATGT	CGTTTGTAGGTGGTGGCTGTG
GADPH	GTCTTCACCACCATGGAGAAG	GTTGTCATGGATGACCTTGGC

Supplementary Table 2. Components of various concentrations of dECM pre-hydrogel mixture

Concentration	SDCM (50 mg/ml)	10× PBS	distilled water	1 M NaOH
10 mg/ml	200 µl	100 µl	680 µl	20 µl
20 mg/ml	400 µl	100 µl	460 µl	40 µl
30 mg/ml	600 µl	100 µl	240 µl	60 µl
40 mg/ml	800 µl	100 µl	20	80 µl

Supplementary Table 3. Primer pairs used in the qRT-PCR.

Gene	Forward primer (5’-3’)	Reverse primer (5’-3’)
iNOS	CAAGCACCTTGGAAGAGGAG	CAAGCACCTTGGAAGAGGAG
CD206	AGACGAAATCCCTGCTACTG	CACCCATTCGAAGGCATTC
TNF-α	AGCCCACGTCGTAGCAAACCAC	AGGTACAACCCATCGGCTGGCA
ARG-1	AGGTACAACCCATCGGCTGGCA	GGAATCTGCATGGGCAACCTGTGT
GADPH	CTCCACTCACGGCAAATTCA	GCCTCACCCCATTTGATGTT

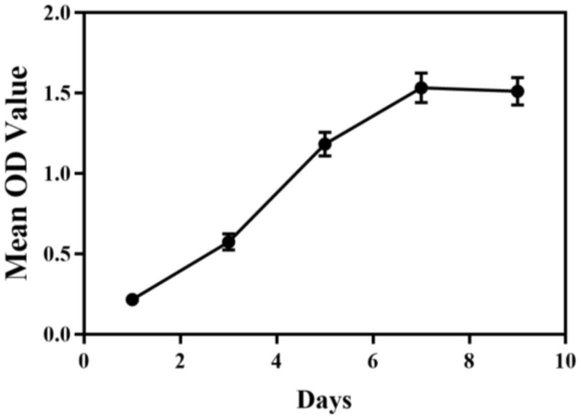

Supplementary Figure 1. Proliferation activity of the USCs as determined by a CCK-8 assay after 1, 3-, 5-, 7- and 9-days' incubation， n = 4.

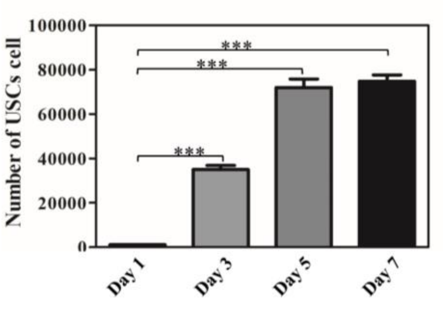

Supplementary Figure 2. Quantitation of USCs using the CyQUANT® Cell Proliferation Assay Kit. *$P < 0.05$, **$P < 0.01$, ***$P < 0.001$, * is the statistical difference compare with Day 1 group. One-way ANOVA followed by Tukey post hoc test was used. Each data point represented average ± standard deviation， n = 4.

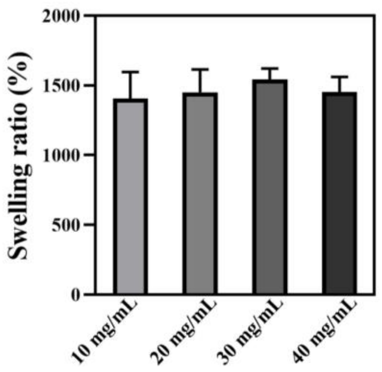

Supplementary Figure 3. Swelling behavior of the dECM hydrogels soaked in PBS， n = 4.

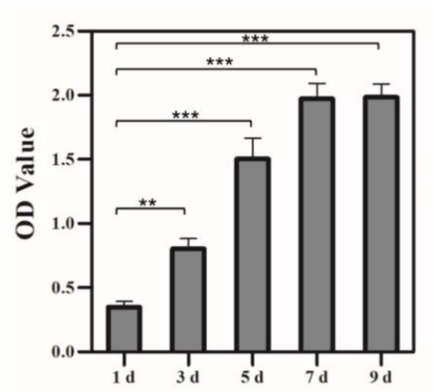

Supplementary Figure 4. Proliferation of the USCs cultured in dECM hydrogels as determined by CCK-8 assay after 1, 3-, 5-, 7- and 9-days' incubation. ns, not significant; *P < 0.05, **P < 0.01, ***P < 0.001, * is the statistical difference compare with 1d group. One-way ANOVA followed by Tukey post hoc test was used. Each data point represented average ± standard deviation， n = 4.

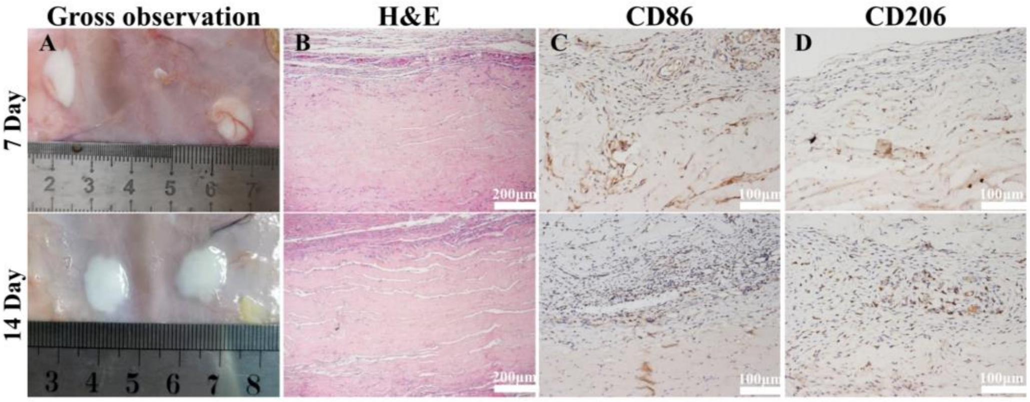

Supplementary Figure 5. Histological analysis of the dECM hydrogels and surrounding tissues at various time points following subcutaneous injection in rats. (A) Gross appearance of the dECM hydrogels at 7 and 14 days. (B) Representative images of H&E staining of the dECM hydrogels at 7 and 14 days after the injection. (C-D) Immunofluorescence of CD86 and CD206 expression in the hydrogels at 7 and 14 days after injection (Scale bar = 200 μm).

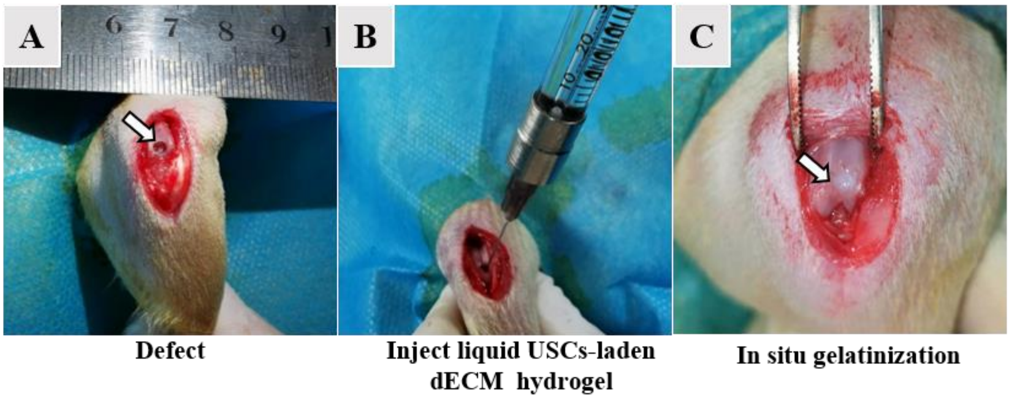

Supplementary Figure 6. The cartilage defect animal model and hydrogel injection. (A) The full-thickness cartilage defect model was made; (B) The USCs-laden dECM hydrogel was injected into the cartilage defect in the patellar groove of distal femur; (C)The hydrogel in situ gelatinization after injection.

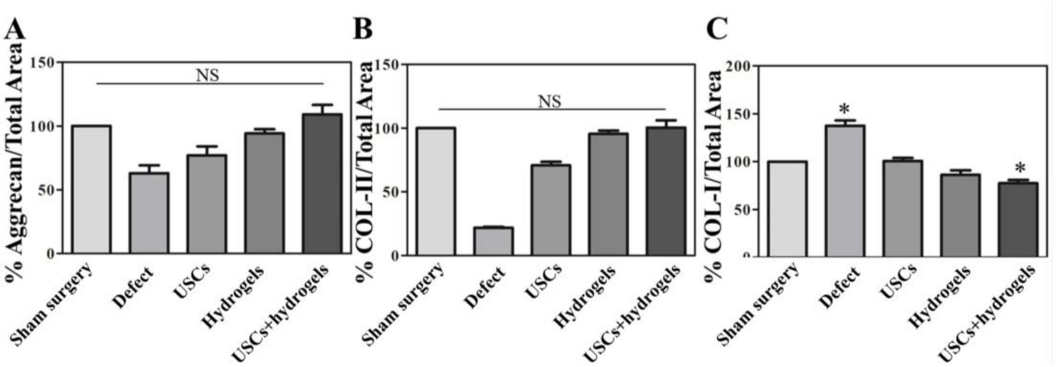

Supplementary Figure 7. Quantitative analysis of aggrecan, collagen II and collagen I immunochemistry staining. (A) Percentage of Aggrecan positive area relative to the total area of each group as compared after 12 weeks. (B) Percentage of COL-II positive area relative to the total area of each group as compared after 12 weeks. (C) Percentage of COL-I positive area relative to the total area of each group as compared after 12 weeks. ns, not significant; **P* < 0.05, ***P* < 0.01, ****P* < 0.001, * is the statistical difference compare with Sham surgery group. One-way ANOVA followed by Tukey post hoc test was used. Data was presented as mean ± S.D. n = 4.

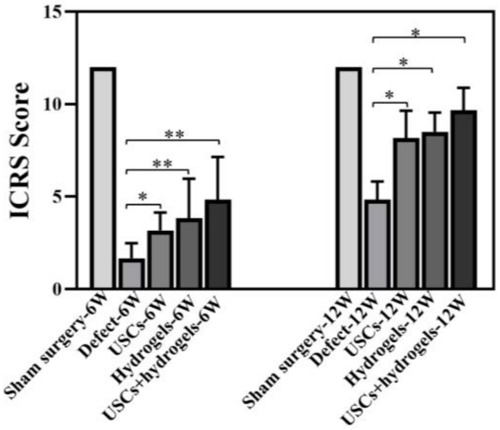

Supplementary Figure 8. ICRS score grading of the cartilage defects. ns, not significant; **P* < 0.05, ***P* < 0.01, ****P* < 0.001, * is the statistical difference compare with Sham surgery group. Data were presented as mean ± SD, n = 4.
